# A Triplet Pregnancy with Spontaneous Delivery of a Fetus at Gestational Age of 20 Weeks and Pregnancy Continuation of Two Other Fetuses until Week 33

**DOI:** 10.5539/gjhs.v8n2p88

**Published:** 2015-06-11

**Authors:** Maryam Ghorbani, Somayeh Moghadam

**Affiliations:** 1 Qom University of Medical Sciences, Qom, IR Iran; 2 Alborz University of Medical Sciences, Alborz, IR Iran

**Keywords:** delayed interval delivery, multiple pregnancy, spontaneous abortion

## Abstract

**Introduction:**

The prevalence of pregnancies with triplet or more has been increased due to using assisted reproductive treatments. Meanwhile, multiple pregnancies have higher risks and long-term maternal-fetal complications compared to twin and singleton pregnancies. Delayed interval delivery (DID) is a new approach in the management of multiple pregnancies following delivery or abortion. The purpose of this paper is to evaluate the benefits of DID and presents a case that used this method.

**Methods:**

This paper covers a report on a case of triplet pregnancy resulting from assisted reproductive techniques with spontaneous delivery of a fetus at gestational age of 20 weeks and the use of conservative DID for two other fetuses until the 33^rd^ week.

**Results:**

In our case, the delivery of two other fetuses occurred spontaneously at gestational age of 33 weeks after the delivery of the first fetus at week 20.

**Conclusions:**

Using DID is a useful and reliable method, but requires careful monitoring, especially in patients with a history of infertility.

## 1. Introduction

The prevalence of pregnancies with triplet or more has been increased due to using assisted reproductive treatments and is directly correlated to the number of embryos transferred into the uterus after IVF. In the United States, the incidence of pregnancies with triplet or more reached 184 per 100,000 from 37 between 1980 and 2002. Although a slight decrease was observed in the incidence in 1998 (Papageorghiou, Avgidou, Bakoulas, Sebire, & Nicolaides 2006), the rate of pregnancies with triplet or more began to fall in 1990, but the rate of twin pregnancies generally increased to 25 percent during this period (Hasson, Shapira, Many, Jaffa, & Har-Toov, 2011).

Multiple pregnancies have higher risks and long-term maternal-fetal complications compared to twin and singleton pregnancies. The two major complications in triplet pregnancies are fetal loss before 24 weeks and early preterm delivery before 32 weeks (Papageorghiou et al., 2006). As all multiple pregnancies are on the rise, the incidence of prematurity and its complications are significantly correlated with the number of embryos (Biard, Bernard, Thomas, & Hobinont 2000). Other complications include increased risk of preeclampsia, hypertension, and premature rupture of membranes compared to singleton pregnancies. In these pregnancies, the risk of prematurity and low birth weight is 10 times, the risk of fetal death is 5 times, neonatal death is 7 times and cerebral palsy is 4 times higher compared to singleton pregnancies. Also twin pregnancy has long-term consequences such as increased risk of neurological complications in future such as epilepsy and learning disabilities. Maternal morbidity is specifically increased due to hypertension, preeclampsia, thromboembolism, urinary tract infection, placental abruption, placenta previa and maternal mortality. Delayed interval delivery (DID) is a new strategy in the management of multiple pregnancies following delivery or abortion. This approach was first introduced by Carson in 1880. This technique is sometimes suggested in elective multiple pregnancies in which uterine contractions cease after the abortion of the first fetus. This method can be an appropriate technique to manage the remaining embryos. It seeks to avoid termination of pregnancy and aims to keep fetuses until they are viable (Ismail Temur, 2013). There is no exact evidence due to the use of antibiotics, tocolysis, or cerclage in prolonging multiple pregnancies. When there are no signs of infection and membranes are intact, prophylactic antibiotics are controversial. In all cases with interval management delivery antibiotic prophylaxis continued. But in some reports they use only in the presence of bacterial colonization of the amniotic fluid. Tocolysis has been used after delivery of the first twin, with different combinations, including β-2agonists, calcium channel blockers, anti-inflammatory drugs, MgSO_4_, and progesterone. Using cerclage in interval management delivery is not consensus. The invasive nature of cerclage and the increased risk of chorioamnionitis due to the closure of a potentially infected amniotic sac is certainly a great concern. Once such a procedure had been done, strict bed rest in the hospital was suggested. Infection, abruption, fetal demise, and fetal anomaly must be recognized and treated appropriately. But after delivery of the first fetus, for delaying the delivery of subsequent fetuses, therapy should be started. Although there is no conclusive evidence supporting the antibiotics, cerclage, and tocolysis should be used for improveing the outcome of the remaining fetuses (Kao, Hsu, & Ding, 2006). A number of case reports show that DID of the second embryo or other embryos is a practical approach in some cases and this approach can lead to a reduction in perinatal mortality (Zhang, Martin, & Trumble, 2004). Therefore, due to controversies in applying this approach, further studies are needed to evaluate the effectiveness of this method in case reports and this paper is presented with the aim of presenting a report on the successful application of this method.

## 2. Case Report

A 22-year-old gravid woman with a triplet pregnancy of 20±3 weeks was admitted to Hazrat Zahra Hospital, Qom on March 2014. Vaginal examination performed on admission showed an open cervix and the amniotic sac inside the vagina. Patient’s history revealed that she had been married for about 3 years and got pregnant with IUI treatment due to infertility for a year. She was cerclaged in early pregnancy and had gestational diabetes. Upon admission, ampicillin was administered intravenously, and ringer serum plus hyoscine were administered to relieve pain. Due to patient’s increased pains and the amniotic sac inside the vagina, cerclage was removed and she was injected with 50 mg pethidine intramuscularly. About 6 hours later, vaginal examination was performed, when limbs of the first fetus could be touched inside the cervix. The first fetus began to expel at 9:25 a.m., and her hand could be observed out of the vagina. The first baby girl was born with Apgar score of 0.0 at 14:50. But, the placenta was not expelled and umbilical cord was visible out of the vagina. On the same day, as the placenta was not expelled, 2 grams ampicillin was injected in umbilical arteries, but the placenta was not expelled. The patient and her husband did not consent to the use of oxytocin and about 2 hours later, the patient was transferred to the midwifery ward to rest. About an hour later, the expelled umbilical cord was spontaneously separated, but the placenta was not expelled. On March 21, 2014 the following report was presented by ultrasound of the fetuses: the right fetus has variable presentation, normal amniotic fluid, and a posterior placenta at gestational age of 20 weeks and 6 days and the left fetus has variable presentation, normal amniotic fluid, and a posterior placenta at gestational age of 20 weeks and 3 days. A diamniotic dichorionic pregnancy is confirmed. Cervical canal length was about 41 mm, and its os was open. A heterogeneous area with dimensions of 48 × 28 is observed in lower part of the uterus near internal os that might be the aborted placenta. Pregnancy ultrasound was again performed on March 27, 2014 and it was reported that both cephalic fetuses were male with normal amniotic fluid and both placentas had right lateral posterior position at 21-22 weeks gestation. The expelled placenta was not observed yet. The patient was hospitalized and monitored for about 2 weeks in the midwifery ward and then discharged against medical advice with good general condition and without vaginal bleeding. During the hospitalization, she was checked every other day for CBC, diff, ESR and CRP tests which were all within the normal range. During pregnancy, the patient received 40 mg clexane subcutaneously. About 90 days later she was readmitted on June, 2014 at gestational age of 33± 4weeks due to the beginning of pain and uterine contractions, and vaginal examination showed that the cervix was dilated 9 cm. So emergency cesarean section was performed to deliver fetuses who had good Apgar scores and were transferred to the NICU.

## 3. Discussion and Conclusion

In a study conducted by Temur in 2013, a case of DID was presented as follows:

The case was a 31-year-old pregnant woman with 8 years past her first pregnancy and primary infertility. Her husband had oligoasthenospermia. The patient was pregnant with twins after ICSI. Her pregnancy was not high risk until 16 weeks, but the first fetus was aborted spontaneously at 17 weeks and 6 days without abortion symptoms (pain, bleeding, and abdominal cramping). The first fetus was born dead and premature rupture of membranes was observed just before the abortion. The aborted fetus had no major abnormalities. After abortion, the health of the second fetus was examined by ultrasound. It had a normal amount of amniotic fluid and a normal gestational sac. The cervix length was reduced to less than 25 mm; internal os was open with dimensions of 10 × 15mm, but external os was closed. According to the results, after reviewing all interventions (cerclage, tocolysis, bed rest, prolonged hospitalization and antibiotic therapy) and discussing risky complications (maternal and neonatal sepsis, severe bleeding, the need for hysterectomy and risk of death), the patient was advised about all treatment options and a consent form was obtained from her. Under general anesthesia in the operating room, the umbilical cord was cut as closely as possible to internal os and the placenta was left in the uterus and McDonald cerclage was performed. The patient was locally and generally monitored for infection, bleeding and coagulopathy disorders. On the day of surgery, the patient was checked for infection and the results were as follows: body temperature of 37.5 °C, leukocytes 12,000 per cubic millimeter, and CRP ++ (measured qualitatively). Interventions were started as follows: broad-spectrum antibiotic therapy (ceftriaxone sodium 4 gr per day intravenously), tocolysis with ritodrine HCL (150 mg per hour) and clindamycin vaginal cream twice daily for 14 days. CRP was reported ++++ two days later, but it was negative after 5 days. The patient was hospitalized during pregnancy up to 132 days i.e. at 36 weeks and 6 days. All interventions were stopped after 14 days, but the patient was monitored for maternal and fetal infection symptoms, any symptoms of premature delivery and fetal health. CRP level was measured weekly, white blood cells and complete blood count were measured at baseline and then monthly and all were insignificant. A dose of 12 mg of betamethasone was administered to promote lung maturation at 28 and 32 weeks. No anticoagulant therapy was undertaken. The baby was born by cesarean section at 36 weeks and 5 days due to breech position. The first minute Apgar score was 8 and the fifth minute Apgar score was 10 and the baby did not need hospitalization in the NICU. The baby and mother were discharged from the hospital in good health 5 days after delivery. The neonatal development and postpartum period did not have a problem. There were no maternal complications. Mother and baby were discharged in good health 5 days after delivery. The placentas had no histopathological problem. In addition, the patient was pregnant with her second baby without any treatment (Ismail Temur, 2013).

The present study has some differences and similarities with our study, in both studies the placenta was remained in uterus and umbilical cord was cut. But in Temur study the patient was hospitalized all the time between birth of first fetus and second fetus. But in our study, our patient discharged after two weeks with personal consent and readmitted about 90 days later again with uterine contraction.

A quadruplet pregnancy occurred in a 32-year-old female which had primary infertility because of endometriosis. And pregnancy was result of oocyte retrieval and tubal embryo transfer. In second trimester, one fetus reduced by using intracardiac KCl injection. But spontaneous rupture of membranes occurred at 29 weeks of gestation. Tocolytic and prophylactic antibiotic treatment were began for patient. The birth of first fetus occurred four days later and a male fetus was delivered with weight of 1,235 g and Apgar scores of 7 and 9 at 1 and 5 minutes, respectively. There was no evidence of placenta abruption, vaginal bleeding, or contraction of uterine. The treatment with tocolytics, steroids, and antibiotics were continued. Complete blood count and C-reactive protein had normal range. Preterm labor occurred at 31 weeks of gestation. Emergency cesarean section was performed because of breech presentation. The birth weight of two male fetuses was 1,440 and 1,285 g and Apgar scores were 7 and 9 at 1 and 5 minutes, respectively. They were taken to the neonatal intensive care unit for observation. After follow-up visits for 2 years, the first infant had retinopathy while the other 2 infants were healthy without any deficits (Kao et al., 2006).

The present study has some differences with our study. In this study spontaneous rupture of membranes occurred at 29 weeks of gestation but in our study this problem was found in gestational age of 20 weeks. In study done by Kao et al, tocolysis began after birth of first fetus but in our study this way (Except use of Hyoscine) was not selected because physicians tendency in our research was to terminate pregnancy. In our study, placenta of first fetus was remained in cervix and delayed delivery was done spontaneously about 13 weeks later that was an approximately longer time than other current studies. But researchers in current study did not mention any thing about placenta of first fetus and delayed delivery of other fetuses happened about 2 weeks after first delivery.

Petousis et al in 2012 done a study. The goal was examining the efficacy of delaying the delivery of the second fetus with tocolysis and emergency cervical cerclage after delivery of first fetus. They entered 5 cases with dichorionic twin pregnancies after miscarriage of the first fetus (<24 weeks). And they analyzed the obstetric and neonatal outcomes. They concluded emergency cervical cerclage has beneficial effect on obstetric and neonatal outcomes for second fetus. In this study emergency cerclage was applied after birth of first fetus but in our study cervical cerclage was opened due to termination of pregnancy (Petousis et al., 2012).

Several studies have examined the consequences of applying DID as follows:

Rosbergen et al. (2005) investigated short-term and long-term results of twin pregnancies after DID. They reported that DID of the remaining fetus had a positive effect on short-term results i.e. these infants were similar to infants with the same gestational age (Rosbergen et al., 2005).

In the study of Wouter et al. (2009), the first fetus was born at 18 weeks and 6 days and the second fetus was born at 37 weeks and 6 days. Using prophylactic tocolysis with antibiotics and administration of corticosteroids after delivery of the first fetus led to the prolongation of pregnancy more than three months and a term infant was born in year (Wouters, Gianottn, Bayram, & Doornbos, 2009).

Zhang et al. and Oyelese et al. independently conducted two population-based studies using similar data for multiple birth records between 1995 and 1998. Zhang et al. reviewed 200 twin pregnancies in which the first fetus was born between 17 and 29 weeks and the second fetus was born two or more days later. They compared the data obtained with the data of 374 twin pregnancies in which the second fetus was born on the same day or the next day in terms of perinatal and fetal survival outcomes. They concluded that DID of the second fetus for 2 days or more before 30 weeks will lead to improved infant survival (Zhang, Martin, & Trumble 2004). Oyelese et al. conducted a retrospective cohort study in 2005 based on the data of 4257 twin pregnancies between 1995 and 1998, in which the first fetus was delivered vaginally at 22 to 28 weeks and investigated the effect of DID on pregnancy outcomes between the two groups. In the first group, the first fetus was delivered between 22 and 23 weeks and the second one with delay of less than 3 weeks and in the second group, the second fetus was born immediately after the first one. They concluded that DID of the second baby leads to improved infant survival and reduced infant mortality (Oyelese, Ananth, Smulian, & Vintzileos, 2005).

## 4. Conclusion

According to our study and literature review, we conclude that DID is a reasonable strategy needed especially in cases of infertility, especially if patients are monitored carefully and accurately.

Almost in all reported cases, treatment strategies for the first fetus included cutting the umbilical cord in the internal os, using broad-spectrum oral and intravenous antibiotics, leaving the placenta of the first fetus inside the uterus, and bed rest for a specific time or throughout pregnancy. However, in most cases, the use of cerclage is still controversial and it is used less than other methods.

## References

[ref1] Biard J. M, Bernard P, Thomas K, Hobinont C (2000). Conservative management of triplet pregnancy after delivery of one foetus.

[ref2] Hasson J, Shapira A, Many A, Jaffa A, Har-Toov J (2011). Reduction of twin pregnancy to singleton: Does it improve pregnancy outcome?. Early Online.

[ref3] Kao S. P, Hsu S, Ding D. C (2006). Delayed interval delivery in a triplet pregnancy. Chin Med Assoc.

[ref4] Oyelese Y, Ananth C. V, Smulian J. C, Vintzileos A. M (2005). Delayed interval delivery in twin pregnancies in the United States: Impact on perinatal mortality and morbidity. Am J Obstet Gynecol.

[ref5] Papageorghiou A. T, Avgidou K, Bakoulas V, Sebire N. J, Nicolaides K. H (2006). Risks of miscarriage and early preterm birth in trichorionic triplet pregnancies with embryo reduction versus expectant management. New Data and Systematic Review.

[ref6] Petousis S, Goutzioulis A, Margioula-Siarkou C, Katsamagkas T, Kalogiannidis I, Agorastos T (2012). Emergency cervical cerclage after miscarriage of the first fetus in dichorionic twin pregnancies: Obstetric and neonatal outcomes of delayed delivery interval. Arch Gynecol Obstet.

[ref7] Rosbergen M, Vogt H. P, Baerts W, Eyck J. V, Arabin B, Marjolein J. M, Lingen V (2005). Long-term and short term outcome after delayed-interval delivery in multi-fetal pregnancies. Eur J Obstet Gynecology Reprod Biol.

[ref8] Temur I (2013). A Twin Pregnancy Provided with ICSI, an abortion of one first fetus at 18th week and live birth of the second fetus at the end of the 36 week. A case report and literature review.

[ref9] Wouters K. A, Gianottn J, Bayram N, Doornbos J. P. R (2009). Term life birth after late abortion of first twin. Acta Obstet Gynecol.

[ref10] Zhang J, Martin B, Trumble J (2004). Delayed interval delivery and infant survival: A population-base study. Am J Obstet Gynecol.

